# Community Perspectives on Access to and Availability of Healthy Food in Rural, Low-Resource, Latino Communities

**DOI:** 10.5888/pcd13.160250

**Published:** 2016-12-15

**Authors:** Zulema Valdez, A. Susana Ramírez, Erendira Estrada, Kathleen Grassi, Stephanie Nathan

**Affiliations:** 1 University of California, Merced, California; 2 Merced County Department of Public Health, Merced, California.

## Abstract

**Introduction:**

Attention has focused on the food environment as a result of the growing concern with obesity rates among Latinos in rural areas. Researchers have observed associations between a lack of physical access to affordable produce in areas where supermarkets and grocery stores are limited and poor dietary intake and obesity; these associations are high in rural, low-resource neighborhoods with a high population of Latino residents. We aimed to engage residents of low-resource, Latino-majority neighborhoods in discussions of food access in a rural yet agricultural community setting, which is typically described as a “food desert.”

**Methods:**

We used a mixed-methods approach and conducted 3 focus groups (n = 20) and in-depth interviews (n = 59) and surveys (n = 79) with residents of a rural yet agricultural community. We used thematic analysis to explore residents’ perceptions of access to healthy foods.

**Results:**

Residents (n = 79; mean age, 41.6 y; 72% female; 79% Latino; 53% Spanish-speaking) reported that dollar and discount stores in this agricultural area provided access to produce; however, produce at retail stores was less affordable than produce at nonretail outlets such as fruit and vegetable stands. Gifts and trades of fruits and vegetables from neighbors and community organizations supplied no-cost or low-cost healthy foods. Residents’ suggestions to improve food access centered on lowering the cost of produce in existing retail outlets and seeking out nonretail outlets.

**Conclusion:**

Our findings contribute to understanding of the food environment in low-resource, rural yet agricultural areas. Although such areas are characterized as “food deserts,” residents identified nonretail outlets as a viable source of affordable produce, while indicating that the cost of retail produce was a concern. Innovative policy solutions to increase healthy food consumption must focus on affordability as well as accessibility, and consider alternate, nonretail food outlets in agricultural areas.

## Introduction

The neighborhood food environment shapes the dietary patterns of its residents (1). In rural, low-resource, Latino-majority neighborhoods, there are few chain supermarkets or grocery stores (2,3). In the absence of traditional food outlets, residents rely on nontraditional outlets, such as discount stores, which tend to stock a limited supply of healthy food at higher prices (4–7). This is especially the case in rural areas, where grocery stores are fewer and farther between, as large food companies merge to remain competitive (8,9). Lack of access to healthy produce by food retailers, and affordability or cost of produce within those retail outlets (10), influences dietary intake by limiting residents’ consumption of fruits and vegetables; this relationship is linked to diet-related behaviors associated with obesity and preventable chronic disease (11,12).

Research has identified barriers to food access and established an association between food access and health disparities in rural, low-resource communities, but understanding of this association is incomplete (6). Missing from this discussion are the experiences and views of the residents themselves (13); what their perceptions of the food environment are and whether they confirm disparities in food access and affordability is not well understood. Also overlooked are residents’ strategies and solutions to improve food access in their specific contexts (for an exception, see Carnahan et al [14]). In keeping with an assets-focused approach (14), which seeks to identify resources for and solutions to problems of food access, and a culture-centered approach (15), which centers the insights of community members within their neighborhood context, we aimed to understand the experiences of residents of rural, low-resource, Latino-majority neighborhoods and solicited their input on strategies to improve the food environment. The distribution of US Latinos in rural areas is large and growing, especially in the nonmetropolitan West where 1 of 5 rural Latinos reside (16). Our study draws attention to the difficulties associated with food access for this understudied population in a rural yet agricultural setting.

## Methods

We used a community-engaged approach to recruit participants from a rural county in Central California as part of a larger chronic disease prevention communication campaign for the county. The concurrent mixed-methods approach (12) included semistructured interviews (n = 59), 3 focus groups (n = 20), and closed-ended surveys (n = 79). Interview and focus group guides (Appendix) had identical questions, with one exception unrelated to the data reported in this article. The guides were developed using an assets-focused approach (14), with the aim of identifying solutions on the basis of how residents characterized their communities’ strengths “on the ground.” The survey questionnaire included previously validated standardized measures of perceptions of food access and neighborhood quality, health beliefs, and demographic characteristics (17) and was pilot tested for internal consistency and reliability. Interview and focus group data were collected, coded, and analyzed separately and then compared to ensure a triangulated and consistent analysis strategy across the multiple methods to achieve trustworthiness and confirmability.

In August and September 2015, we purposively recruited residents from 2 communities in Merced County, South Merced and Winton. Recruiting sites were 2 flea markets, the local mall, waiting rooms of community health clinics, churches, and health fairs. Recruitment was conducted by trained undergraduate research assistants using both passive recruitment (ie, informational booths at recruitment sites) and active recruitment (ie, solicited potential participants in waiting rooms). Informational flyers that were approved by an institutional review board described the study. Potential participants were screened for the following inclusion criteria: being aged 18 years or older, being a Merced County resident, and able to speak English or Spanish.

Merced County is 1 of 18 counties that comprise the 450-mile agricultural region of Central California (population 6.5 million), where 40% of California’s Latinos reside (16,18). The agricultural industry is the largest industry and employer in Merced County, employing 15% of residents year-round (18,19). According to the California Department of Public Health, 34.1% of Merced County residents are obese, with a higher prevalence of obesity among people who are low-income and Latino than among people of high-income or who are white, although all groups have had marked increases in obesity prevalence since 2003 (20). In 2010, 14.3% of Merced County rural residents lived more than 10 miles from a supermarket or large grocery store, a factor associated with food deserts (21). The US Department of Agriculture also reports that in 2012, convenience stores (n = 74) and fast food restaurants (n = 129) outnumbered grocery stores of any size (n = 58) in Merced County (21). One in 6 (17%) households in Merced County is food insecure (21). Although disparities exist in availability of supermarkets and grocery stores in this rural area when compared with urban areas, discount and dollar stores in California capitalize on the agricultural industry to supply seasonal and even organic produce year-round (22).

We recruited a sample of residents from 2 communities in Merced County: South Merced, which is within the bounds of the county seat, the City of Merced, and Winton, which is an unincorporated community north of the City of Merced. These communities were identified as priority areas by the Merced County Department of Public Health owing to specific health disparities experienced by their residents, particularly diabetes and overweight or obesity.

All participants completed the closed-ended survey following the interview or focus group. Participants received a $25 gift card incentive. Interviews and focus groups were conducted by trained bilingual (English/Spanish), bicultural (Mexican/American) research assistants in convenient locations (eg, at recruitment sites if privacy permitted, public spaces such as coffee shops; focus groups were held in conference rooms of centrally located nonprofit organizations). Interviews and focus groups were digitally audio-recorded and professionally transcribed. Focus groups lasted approximately 90 minutes; interviews lasted approximately 45 minutes. The study protocol was approved by the institutional review board of the University of California, Merced.

Transcripts of interviews, surveys, and focus groups were reviewed by researchers. Spanish language transcripts were analyzed in the original language and translated into English for presentation; bilingual research assistants verified translation for conceptual equivalence. An iterative inductive–deductive approach was used to identify themes observed in the data about community members’ perceptions of the food environment, specifically access to and affordability of fruits and vegetables and strategies and solutions to improve the food environment. These themes informed the development of a set of codes for retrieval and selection of text for analysis and interpretation using the qualitative software program ATLAS.ti (Scientific Software Development, GmbH). The analysis of interview data reached a point of saturation at 25 interviews; focus group data provided more detailed descriptions or deeper discussions of common themes than did interview data; survey data were used to report frequencies for key variables. Researchers discussed preliminary findings to establish themes and to select representative interpretive memos and narratives.

## Results

### Participant characteristics

Sample participants were largely representative of the 2 communities. More than half of respondents completed the interview in Spanish, and 79% identified as Latino, a percentage slightly higher than their percentage in Merced County but approximately equal to the proportion in the 2 sampled neighborhoods (Table 1). Sixty-one percent of the participants were born in Mexico. Almost three-quarters of participants were women, one-quarter higher than that of Merced County as a whole (49.5%). Mean age of participants was 42 years, and more than one-quarter were aged 18 to 29 years. Nearly all participants (96%) had children aged 17 years or younger in their household. The socioeconomic position of the predominantly female participants was similar to that of people in the community: only 42% were employed full time, and more than one-third were unemployed; 30% had completed only elementary school, and just under one-quarter had some college or technical training; 60% reported having received food assistance in the previous year. The lack of educational attainment was reflected in low English proficiency; 60% of participants reported speaking English less than “very well” (data not shown).

**Table 1:** Characteristics of Participants (N = 79) in Study of Community Perspectives on Access to Healthy Food in Rural, Low-Resource, Latino Communities, Merced County, California, 2015

Characteristics	Value^a^
**Mean age, y (SD)**	41.6 (14.5)
18–29	27.4
30–44	30.1
45–54	20.6
≥55	21.9
**Sex**	
Female	72.2
Male	27.9
**Race/ethnicity**	
Latino	78.5
White	10.1
Asian	4.0
Black	2.3
Other	5.1
**Speaks Spanish**	53.0
**Country of birth**	
United States	37.7
Mexico	61.0
Other	1.3
**Education, highest level**	
Elementary school	29.9
Grades 7–8	14.3
High school diploma or GED	32.5
Some college or technical training	23.4
**Employment status**	
Full time	42.1
Part time	19.7
Not employed	38.2
**Received food assistance in past year**	59.5
**Number of people in household (SD)**	4.6 (2.1)
**Have children aged 0–17 y in household**	96.2

Abbreviations: GED, general educational development; SD, standard deviation.

a Values expressed as percentages, unless otherwise indicated.

### Access to fruits and vegetables

Most residents in this agricultural setting, where a variety of produce is grown year-round (18,19), reported ample access to produce in their neighborhoods (Table 2). More than 70% of survey respondents agreed with the statement, “A large selection of fruits and vegetables is available in my neighborhood.” This finding contrasted favorably with the smaller percentage of respondents who disagreed (15.4%). Moreover, 62.8% of our survey respondents agreed with the statement, “The fruits and vegetables in my neighborhood are of high quality.” Respondents who participated in focus groups and interviews further emphasized the ease of access to high-quality fruits and vegetables in their communities (Table 3). For example, 2 female focus group participants (aged 64 and 46) agreed that the best aspect of living in their neighborhood was the abundance and quality of the fruit.

**Table 2:** Respondents’ Perceptions about Access to and Affordability of Healthful Foods, Survey (N = 79), Merced County, California, 2015

Perception	Agree	Neutral	Disagree
	%		
A large selection of fruits and vegetables is available in my neighborhood.	70.5	14.1	15.4
The fruits and vegetables in my neighborhood are of high quality.	62.8	21.8	15.4
Lack of access to food shopping is a problem in my neighborhood.	30.4	21.5	48.1
Healthy food options such as fruit and vegetables are too expensive.	64.6	13.9	21.5

**Table 3:** Sample Quotations on Access to and Affordability of Healthy Food and Strategies and Challenges to Improve It, Focus Group Respondents (3, n = 20), Merced County, California, 2015

Theme	Respondent’s Sex/Age in Years	Sample Quotation
Access to healthy food	Female, 46	“[The Dollar Store]…[has] a lot of nice produce.”
	Male, 55	“We have a lot of people . . . they grow . . . work with fruit . . . whenever they got something extra, they’ll pass it around the neighborhood.”

Healthy food affordability	Male, 55	“It would be nice to be able to have a store that you can actually afford to go to.”

Strategies to improve healthy food access/affordability	Female, 61	“I’ve been checking out the [fruit/veg] stands . . . that’s actually been cheaper than it has been at the stores for the quantity you get and the price.”

Challenges to improve healthy food access/affordability	Female, 56	“If I can go to McDonald’s and I can get a chicken sandwich for a dollar and a salad for six dollars I’m going to have to think twice about [choosing the healthy option].”

With regard to retail and nonretail food environments (ie, retailing outside of a fixed or traditional facility [eg, fruit and vegetable stands]), fewer than one-third of survey respondents indicated that a “lack of access to food shopping is a problem in my neighborhood” (Table 2). Respondents suggested that several discount or dollar stores provided sufficient access to produce (Table 3). One female participant (age 46) explained that, “[The dollar store] . . . [has] a lot of nice produce.” Likewise, one male participant (age 25) stated, “You can buy vegetables all over the place.”

Moreover, residents underscored the influence of the agricultural industry in increasing access to produce via alternate, nonretail food outlets. Specifically, a strong agricultural industry presence was noted by participants, evidenced by fruit and vegetable stands staffed by farmers and workers (on farms, roadsides, and swap meets) as well as mobile fruit vendors, and participants noted that gifts of fruit and vegetables were shared among neighbors. As a 55-year-old white male focus group participant stated, “We have a lot of people . . . they grow . . . work with fruit. . . . Whenever they got something extra, they’ll pass it around the neighborhood.” Similarly, 2 female participants (aged 73 and 23) reported that they frequently shared fruits and vegetables grown in their private gardens, backyards, and the local community garden, with “the elderly” and neighbors “across the street.”

### Affordability of fruits and vegetables

Most survey respondents suggested that physical access to quality produce was not a problem in their communities; however, most (65%) reported that “healthy food options like fruits and vegetables are too expensive” (Table 2). Respondents’ concerns about affordability were mostly limited to retail stores, indicating that the differences between our findings and CHIS may stem from this distinction. As a male participant noted, “It would be nice to be able to have a store that you can actually afford to go to . . . even the dollar store isn’t the dollar store anymore.” Respondents suggested that fruit and vegetable stands and flea markets, as opposed to retail stores, were good sources for cheap produce.

### Strategies and solutions to improve food access and affordability

Respondents indicated that physical access to healthy food was not a problem. Although there were fewer supermarkets and grocery stores in this rural, low-resource area than outside it, alternate food outlets such as dollar stores, fruit and vegetable stands, and neighbors combined to ensure an abundance of accessible produce. Nevertheless, residents indicated concerns about the cost of retail produce. To improve affordability, respondents suggested that residents do their research, look for sales, and comparison shop. Others advised residents to avoid retail stores altogether in favor of nonretail food outlets. As one female participant (age 61) stated, “I’ve been checking out the [fruit and vegetable] stands. . . . That’s actually been cheaper than it has been at the stores for the quantity you get and the price. And you can’t get it any fresher.” Still others called for the local business community and government to encourage the development of new food outlets and healthy restaurants. Several interviewees observed positive changes in existing settings that they hoped would continue, such as replacing energy-dense snacks with fruit in the school lunch program.

Rather than focusing on access and affordability of healthy food, some residents maintained that the problem of better health through dietary intake rested on the access to cheap fast food. Consistent with research extending the metaphor of “food desert” to “food swamp” (23), residents reported the profusion of fast-food restaurants in the area, which outnumber grocery stores 2 to 1. Many residents believed that easy access to cheap, fast food was a bigger threat to healthy food intake than access to healthy food, and made direct zero-sum comparisons to that effect. As a female participant (age 56) explained, “If I can go to McDonald’s and I can get a chicken sandwich for a dollar and a salad for $6, I’m going to have to think twice about [choosing the healthy option].” Likewise, a male participant (age 20) stated, “It is much cheaper to buy, like, unhealthy food and a larger quantity of that to feed a family on a lower income than it is to buy healthier food with the same amount of money.”

## Discussion

In a departure from previous research findings, our findings indicate that in the vast agricultural region of Central California, Latino-majority residents of traditionally defined “food deserts” did not report a lack of access to healthy produce. In the absence of supermarkets and grocery stores, respondents relied on alternate food outlets, specifically discount stores, fruit and vegetable stands, and gifts of produce from neighbors in this agricultural area. Although research on food access tends to focus on physical access, previous research indicates that cost is a major barrier to healthy food consumption in rural areas (24,25). In keeping with this research (3), respondents emphasized a dearth of affordable healthy food in retail stores. Perceptions of barriers to healthful eating — in this case, relating to access and cost — are important, because research demonstrates that they may be more powerful predictors of health than objective barriers (26,27). Community members’ solutions and strategies to improve the food environment centered on calls to increase affordability of retail produce, seek out alternate nonretail outlets, and increase healthy food in existing settings (eg, school lunch).

Although our survey was based on a small convenience sample (n = 79), findings on access to fruits and vegetables were consistent with those of the larger 2014 California Health Interview Survey (CHIS) (28). More than 70% of our survey respondents agreed with the statement, “A large selection of fruits and vegetables is available in my neighborhood.” According to CHIS, approximately 77% of Merced County residents agreed that they could “always” find fresh produce in their neighborhood. However, our findings on affordability of fruits and vegetables differed somewhat from those of the 2014 CHIS. Most of our survey respondents suggested that options for fruits and vegetables were too expensive in their communities. Less than one-third (31%) of Merced County respondents of the 2014 CHIS indicated that fresh produce was never affordable or only sometimes affordable (21).

Our study has several limitations. First, data were from a nonprobability convenience sample for reasons of cost and time. Although our sample was diverse across several demographic characteristics, it overrepresented nonworking women; this factor limits the generalizability of our results. Moreover, our research instruments did not include questions on occupation or industry; in retrospect, participants’ relationship to the agricultural industry would have informed our understanding of its centrality to the food environment. Nevertheless, our research suggests that the agricultural industry increased access and affordability of produce in “food deserts” via retail outlets such as dollar stores and alternate outlets such as fruit and vegetable stands. Incorporating these insights into future research will help to clarify this relationship.

Our findings suggest that a culture-centered approach (29) to understanding food environments and their impact on health and health disparities indicates nuances in thinking about the problem and generates original solutions rooted in specific communities and neighborhood contexts. Innovative policy solutions and interventions aimed at increasing healthy food access as a strategy for obesity and chronic disease prevention must focus on affordability as well as availability and consider alternate food outlets in agricultural areas.

## Appendix. Focus Group/Interview Script

First we want to know how you feel about the community where you live.

1. If you could describe Merced County in one word, what would it be?
  - What would you like it to be?
2. In order of importance (with most important at the top), arrange the cards with individual words on each [all cards have an individual letter a–n so that the arranged list can be recorded by interviewer], describing different aspects of a neighborhood you feel are important to enable you to live a healthy, happy life.
  - Safe
  - Healthy
  - Community
  - Nutrition
  - Education
  - Environment
  - Wellness
  - Cleanliness
  - Caring
  - Exercise
  - Employment
  - Amenities
  - Shopping
  - Infrastructure
  - Are there any other words you would like to add?
3. What do you see as the best aspect of Merced County [your neighborhood]?
4. What do you feel is the worst aspect of Merced County?
5. Do you enjoy living in Merced County [your neighborhood]?
  - Why? How does it impact your life?
6. If you could change anything about Merced County [your neighborhood], what would it be?
  - Why?
7. Do you feel that Merced County [your neighborhood] is generally safe?
  - What makes you think that?
  - What does the word “safe” mean to you?
8. What does the word “healthy” mean to you?
9. Do you feel that Merced County [your neighborhood] is generally healthy?
  - What makes you think that?
10. What do you think are the major health issues in Merced County?
11. What do you think can actually be done to improve the health of Merced County residents?
  - Who could be responsible for making these changes?
12. Do you feel that Merced County officials understand the challenges that people in your neighborhood face?
  - Now we are going to switch gears and talk about where you get information.
13. What is the best place to get news and information about what is going on around your local neighborhood or area?
14. What do you consider to be the most reliable and trustworthy source of information that might influence your thoughts or behavior?
  - Lastly, we are going to ask you questions about the media. Think about all of the different commercials or ads you have seen in the last 12 months, for any products, places, or ideas.
15. Of all the ads or commercials you might have seen in the past 12 months, which ads come to mind right away?
  - What do you remember about them?
16. Think about a poster showing Merced County. What image would you like to see representing Merced County?
  - What do you think the poster would look like in reality, based on living here in Merced County?
17. San Diego uses the phrase “Live Well” to promote itself as Healthy, Safe and Thriving. [SHOW IMAGE OF SD LIVE WELL] What are your opinions of this approach/idea?
18. If you were asked to help improve the health of Merced County [your neighborhood] where would you start?
19. What comes to mind when you hear the following words:
  - Health
  - Safety
  - Happiness
  - Community
  - Thriving
  - (Other adjectives can be added here)
20. Do you have any suggestions about how Merced could change in order for you to live a better life that we have not asked you about?
